# Maternal control of children’s daily toothbrushing: a cross-sectional study

**DOI:** 10.1007/s40368-026-01223-w

**Published:** 2026-05-20

**Authors:** Y. Peneluc Rocha, V. Venske Pinheiro, J. Lima do Amaral, C. de Oliveira Langlois, A. Emidio Ribeiro Silva

**Affiliations:** Postgraduate Program in Dentistry, Federal University of Pelotas, 457 Gonçalves Chaves Street, 5th floor, Rio Grande do Sul, Pelotas, Brazil

**Keywords:** Child, Mothers, Distress, Toothbrushing, Dietary

## Abstract

**Purpose:**

The aim of the present study was to identify the prevalence of lack of maternal control of the daily toothbrushing of children up to 6 years of age receiving care at a School of Dentistry in Southern Brazil, as well as its associated factors.

**Methods:**

A cross-sectional study. Data were obtained through a structured questionnaire administered to the mothers. The outcome of interest was maternal control of their children’s daily toothbrushing, and the exposure variables were related to both the mother and the child. Descriptive and multiple regression analyses were performed.

**Results:**

A total of 156 mother-child pairs were evaluated. The percentage of children whose daily toothbrushing was not controlled by their mothers was 14.8%. After adjusted regression analysis, lack of dietary control (PR = 2.74; 95% CI 1.15–6.50; *p* = 0.023) and maternal stress symptoms (PR = 3.74; 95% CI 1.56–8.96; *p* = 0.003) remained associated with the outcome.

**Conclusion:**

The percentage of children whose daily toothbrushing was not controlled by their mothers was 14.8%. Factors, such as the presence of maternal stress symptoms and the absence of dietary control for the child, directly influenced the lack of maternal control of children’s daily toothbrushing.

## Introduction

Oral health is one of the main indicators of well-being and quality of life in the general population, with dental caries being one of the most prevalent diseases worldwide (Li et al. 2025). In childhood, oral health care is essential for healthy child development (Watt et al. 2024). Factors commonly associated with adverse oral health outcomes in children include a sugar-rich diet, low socioeconomic status, and the absence of parental or caregiver assistance during toothbrushing (Jain et al. 2018; Castilho et al. 2025).

The repercussions of oral diseases in childhood go beyond physical aspects, extending to psychological and social dimensions. Children with dental caries may experience sleep disturbances, pain, discomfort while chewing, and speech impairments (Pakkhesal et al. 2021; Abanto et al. 2011). Evidence from a systematic review and meta-analysis indicated that children aged 3 to 12 years with early childhood caries have a higher likelihood of reporting an impact on oral-health-related quality of life when compared with caries-free children (Moghaddam et al. 2020).

During childhood, parents and caregivers play a crucial role in children’s oral health, as the presence of parental assistance during toothbrushing is associated with a reduced risk of dental caries development (Castilho et al. 2025). This is because many children face motor difficulties during daily toothbrushing due to their age, as well as a lack of motivation for this activity (Grossman and Proskin 1997).

In this context, the role of mothers stands out as they often feel more responsible for their children’s care (Balasooriyan et al. 2024). This literature demonstrates a direct association between mothers’ oral health and their children’s oral health, reinforcing the influence of positive maternal behaviors on the establishment of healthy habits during the child’s early years (Silva et al. 2024). This occurs because, during the initial stages of socialization, children tend to imitate behaviors practiced by their parents—particularly their mothers, who typically spend more time with them and therefore play a central role in shaping and developing health-related behaviors (Snell et al. 2019).

Therefore, maternal knowledge and attitudes regarding oral health have a direct impact on their children’s oral health (Marandi et al. 2025). In light of this, the aim of the present study was to identify the percentage of children up to 6 years of age without maternal control of daily toothbrushing receiving care at a School of Dentistry in Southern Brazil, as well as other related behaviors. This study’s hypothesis is that poorer maternal and child sociodemographic conditions, unfavorable child behavioral factors, and poorer maternal mental health are associated with lower control over the child’s toothbrushing.

## Methods

The present study is reported in accordance with the STROBE guidelines.

### Study design, population and sample

The present transversal study used a non-probabilistic sample of children aged 0 to 6 years who were receiving care at the pediatric dentistry clinics of the School of Dentistry at UFPel, in the city of Pelotas, Rio Grande do Sul, Brazil, between May and October 2024.

### Inclusion and exclusion criteria

All mothers who were waiting with their children in the dental clinic reception area were included and invited to participate. Those who agreed were taken to a private room, where the informed consent and assent forms were read and signed prior to the interview. Children up to 6 years of age who, at the time of the appointment, were accompanied by other caregivers, as well as those whose mothers refused to complete the questionnaire, were excluded from the study.

### Questionnaire and data collection

Data collection was performed using a structured questionnaire composed of 60 questions related to demographic characteristics of the mothers and children, as well as oral-health-related habits and behaviors of both the mother and the child, and maternal mental health.The questionnaire was administered by trained interviewers.

To pretest the questionnaire and minimize information bias, interviewers underwent standardized training. During this process, they were instructed to administer the questionnaire uniformly and without demonstrating judgment or influencing responses, ensuring greater reliability of the collected data.

With regard to oral health, dental caries examinations were performed in the children by two trained and calibrated dentists. To assess dental caries, the DMFT index (decayed, missing, and filled teeth) and the dmft index (decayed, indicated for extraction, and filled teeth), as recommended by the World Health Organization, were used. For the examinations, procedure gloves, masks, caps, and a sterilized clinical kit containing a dental mirror and a WHO probe were used. Both intra- and inter-examiner kappa values were greater than 0.80.

The covariates analyzed in the present study were related to the mother: age (in years, categorized as 17–27 years and ≥28 years); maternal education (in years of schooling, categorized as ≤8 years, 9–11 years, and ≥12 years); maternal number of children (0, 1, or ≥2); maternal perception of the child’s oral health (originally five categories, grouped as good/very good, fair, and poor/very poor); and maternal stress symptoms assessed using the Depression, Anxiety and Stress Scale—21 (DASS—21) (Vignola and Tucci 2014), categorized as “no stress symptoms” or “with stress symptoms.”

For the child, the variables assessed were: sex (female/male); age in years (categorized as ≤ 3 years and > 3 years); maternal dietary control (yes/no); and dental caries in the primary dentition assessed using the dmft index (“decayed, missing, and filled teeth”), categorized as dmft = 0 and dmft ≥ 1.

The study outcome was maternal control of the child’s daily toothbrushing. Responses were obtained through the question: “Do you control your child’s toothbrushing daily?” with response options “yes” and “no.”

### Data analysis

**Fig. 1:**
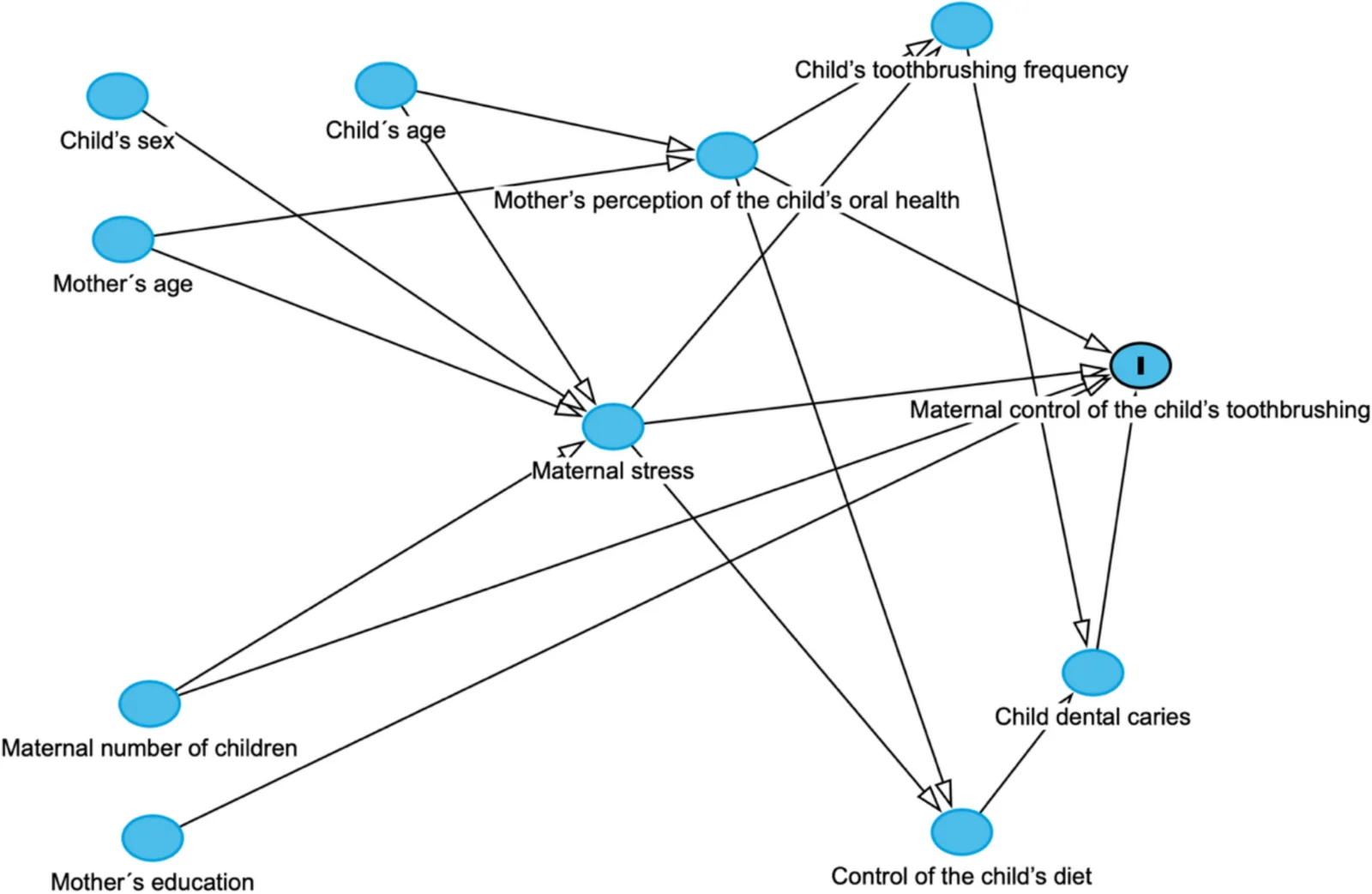
Directed Acyclic Graph (DAG) showing the exposures and outcome (Maternal control of the child’s daily toothbrushing). https://www.dagitty.net

A directed acyclic graph (DAG) was used to identify potential covariates for the analysis of maternal control of the child’s daily toothbrushing (Fig. 1). Data analysis was performed using Stata statistical software. Descriptive analysis was conducted to evaluate absolute and relative frequencies of the sample and of the covariates analyzed in the present in relation to the outcome. Subsequently, Poisson regression analysis was conducted to estimate crude and adjusted prevalence ratios. The adjusted regression model was constructed using the backward method. A significance level of *p* < 0.05 and a 95% confidence interval were considered for all analyses. Due to the low outcome percentage and the limited number of events, the adjusted regression models included only a limited number of variables to avoid overfitting and unstable estimates.

## Results

The total study sample comprised 176 mother/father-child pairs. Of these, 155 were mother-child pairs who participated in the study. The percentage of children whose daily toothbrushing was not controlled by their mothers was 14.8%. Among the participating mothers, most were older than 28 years (70.5%), had 12 or more years of education (35.5%) and 39% had no other children. Regarding the children, most were female (56.8%) and between 3 and 6 years of age (68.8%) (Table 1).

**Table 1:** Descriptive analysis of the study sample and assessment of maternal control of children’s daily toothbrushing according to study variables Brazil, 2026

			Maternal control of the child's daily toothbrushing			
			No		Yes	
	*N*	%	*N*	%	*N*	%
Mother-related characteristics						
Age						
17 to 27 years	46	29.5	7	16.3	36	83.7
Over 28 years	110	70.5	15	14.2	91	85.8
Education						
Up to 8 years	50	32.2	6	12.5	42	87.5
9 to 11 years	50	32.2	5	11.1	40	88.9
More than 12 years	55	35.6	11	20.0	44	80.0
Number of children						
0	60	39.0	11	18.6	48	81.4
1	58	37.6	6	10.5	51	89.5
≥ 2	36	23.4	5	15.2	28	84.8
Perception of the child’s oral health						
Very good/good	81	51.9	11	14.3	66	85.7
Fair	59	37.8	10	17.5	47	82.5
Poor/very poor	16	10.3	1	6.7	14	93.3
Maternal stress						
No stress	99	68.3	7	7,4	88	92.6
With stress	45	31.7	10	22,7	34	72,3
Child-related characteristics						
Sex						
Female	88	56.8	14	16.9	69	83.1
Male	67	43.2	8	12.1	58	87.9
Age						
Children up to 3 years old	48	31.2	6	12.8	41	87.3
Children older than 3 years	106	68.8	15	14.9	86	85.1
Diet control						
Yes	113	76.4	12	11.0	97	89.0
No	36	23.6	10	27.8	26	72.2
Dmft						
0	60	38.6	9	15.8	48	84.2
≥ 1	96	61.5	13	14.3	78	85.7
Toothbrushing frequency						
Up to two brushings	64	42	52	81.2	12	18.8
More than two brushings	89	58	74	88.1	10	11.9

Regarding habits and behaviors related to their children’s oral health, mothers reported that they controlled their child’s diet (75.8%) and perceived their child’s oral health as very good/good (51.9%). Concerning maternal mental health, 41% of the mothers interviewed presented stress symptoms. Finally, regarding the severity of dental caries, 39.5% of the children had a dmft score of 1 or higher (Table 1).

In relation to maternal control of the child’s daily toothbrushing, it was observed that mothers older than 28 years, those who perceived their child’s oral health as poor/very poor, and those with stress symptoms were more likely to report a lack of control (Table 1).

Table 2 presents the analysis of the covariates in relation to the outcome using Poisson regression. In the crude Poisson regression analysis, maternal stress symptoms (PR = 3.01; 95% CI 1.22–7.42; *p* = 0.016), and lack of dietary control (PR = 2.11; 95% CI 1.30–3.45; *p* = 0.015) were associated with the outcome. After the adjusted regression analysis, the same variables remained associated: maternal stress symptoms (PR = 3.74; 95% CI 1.56–8.96; *p* = 0.003) and lack of dietary control (PR = 2.74; 95% CI 1.15–6.50; *p* = 0.023).

**Table 2:** Multiple regression analysis of maternal control over children’s daily toothbrushing according to study variables Brazil 2026

		Maternal control of the child's daily toothbrushing				
		CRUDE			ADJUSTED	
		PR	CI 95%	*p* valor	RP	CI 95%. *p* valor
Mother-related characteristics						
Age						
17 to 27 years		1		0.740		
Over 28 years		0.87	0.38–1.98			
Education						
Up to 8 years		1				
9 to 11 years		0.89	0.29–2.72	0.837		
Over 12 years		1.6	0.63–4.01	0.316		
Number of children						
0		1				
1		0.56	0.22–1.42	0.228		
≥ 2		0.81	0.30–2.14	0.675		
Perception of the child’s oral health						
Very good/good		1				
Fair		1.22	0.55–2.69	0.609		
Poor/very poor		0.46	0.64–3.37	0.450		
Maternal stress						
No stress		1			1	
With stress		3.01	1.22–7.42	0.016	3.74	1.56–8.96 0.003
Child-related characteristics						
Sex						
Female		1				
Male		0.71	0.32–1.61	0.423		
Age						
Up to 3 years old		1				
Older than 3 years		1.16	0.48–2.81	0.737		
Diet control						
Yes		1			1	
No		2.11	1.30–3.45	0.015	2.74	1.15–6.50 0.023
Dmft						
0		1				
≥ 1		0.90	0.41–1.98	0.803		
Toothbrushing frequency						
Up to two		1				
More than two		0.63	0.29–1.37	0.251		

These findings indicate that mothers who reported not controlling their child’s diet had a 174% higher probability of not controlling their child’s daily toothbrushing compared to those who did control their child’s diet. Additionally, mothers identified as having stress symptoms had a 274% higher probability of not controlling their child’s daily toothbrushing compared to mothers without stress symptoms.

## Discussion

The results of this study indicate an association between maternal control of the daily toothbrushing of children under six years of age and both maternal dietary control habits and maternal stress symptoms. Mothers who reported not controlling their children’s diet, as well as those presenting stress symptoms, showed a higher likelihood of not supervising their child’s daily toothbrushing. These findings reinforce the idea that oral health in childhood is associated with maternal psychological factors (Ludovichetti et al. 2023) and with health-related behaviors within the family environment (Liu et al. 2024).

A total of 14.8% of mothers in the present study did not control their child’s daily toothbrushing, a lower percentage than that reported in previous studies assessing parental control, which ranged from 19.5% to 46% (Khan et al. 2021; Zarabadipour et al. 2023). The authors hypothesize that this lower percentage may be due to the fact that these children were already receiving care at a dental school clinic, which facilitates the transmission of information to mothers regarding the importance of controlling daily toothbrushing as a preventive measure against diseases such as dental caries—the most common oral health problem affecting children.

The present study also observed an association between maternal stress symptoms and the lack of control of children’s daily toothbrushing. Mothers with stress symptoms were more likely to not control this practice, reinforcing the influence of maternal mental health on child care (Liu et al. 2024). Maternal overload, stressful work routines, and responsibility for household tasks are factors associated with poorer health among women (Chen et al. 2020; Aviv et al. 2025; Matsuda et al. 2021). One study found that maternal stress may act as a barrier to establishing a toothbrushing routine among children since challenges faced during the process—such as fatigue or tiredness—may render routine parental tasks more taxing (Elison et al. 2014). A possible underlying mechanism for this relationship is maternal self-efficacy, understood as the perception of one’s own ability to positively influence a child’s behavior. Evidence indicates that mothers with higher self-efficacy are more likely to establish consistent oral hygiene routines and to adequately supervise toothbrushing (Finlayson et al. 2007). On the other hand, psychological stress may reduce this sense of control, leading to lower adherence to preventive practices and a weakening of the behavioral structure necessary for maintaining healthy habits (Deater-Deckard 1998).

Thus, the lack of maternal control of daily toothbrushing, as previously described in the literature, may contribute to an increased risk of dental caries during childhood, in the primary dentition, and later in adolescence, in the permanent dentition (Matsuyama et al. 2020).

Previous studies highlight the role of ultra-processed and sugar-rich foods in the development of dental caries, with dietary control being considered an important strategy for preventing and managing the disease (Rocha et al. 2025). Additionally, parental dietary counseling aimed at controlling children’s sugar intake has been associated with a lower prevalence of dental caries (Costa et al. 2022; Pimpin et al. 2013). In this context, mothers who adopt an authoritative parenting style tend to exhibit greater dietary control of their children compared to permissive parents as described in the literature (Quek et al. 2021). Findings from previous studies support the associations observed in the present study, indicating that mothers who do not control their child’s daily toothbrushing are also more likely to have no control over the child’s diet.

Maternal factors, such as educational level and number of children, and child-related factors, including the presence of dental caries, were not associated with the study outcome. More than 80% of the mothers in the present study had more than nine years of schooling. Therefore, it was expected that mothers with higher educational levels would be more likely to control their child’s daily toothbrushing as reported in previous studies (Paramasivam et al. 2025; Bozorgmehr et al. 2013; Dixit et al. 2021). Regarding dental caries, since 14.1% of mothers reported not controlling their child’s daily toothbrushing, the authors of the present study expected a lower prevalence of caries in the primary dentition than the 61% observed, suggesting that a lower prevalence of caries could be associated with greater maternal control. These findings highlight the complexity of the factors involved and underscore the need to explore, beyond sociodemographic characteristics, subjective aspects related to maternal health and the family environment that were not assessed in the present study.

This study presents some limitations that should be considered when interpreting the results. The sample consisted of a convenience sample of mothers and their children aged 0 to 6 years receiving care at a School of Dentistry. In addition, the cariogenic diet variable was assessed using a single question rather than a standardized instrument. Therefore, the findings should be interpreted with caution, particularly regarding their generalizability. Nevertheless, a strength of the study is its focus on the lack of maternal control of children’s daily toothbrushing—a topic that, to the authors’ knowledge, remains underexplored in the literature—highlighting the need for future studies with longitudinal designs to strengthen the scientific evidence on this topic.

## Conclusion

It was concluded that the percentage of children whose daily toothbrushing was not controlled by their mothers was 14.8%. Maternal stress symptoms and the absence of maternal control over the child’s diet were directly associated with the lack of maternal control of the child’s daily toothbrushing. The findings of the present study reinforce the relevance of addressing aspects of maternal mental health and maternal behaviors in child care, given their important influence on the child’s oral health. In light of these results, dental professionals may consider using mental health screening questionnaires for mothers at the beginning of the child’s treatment and, when necessary, referring mothers to health services for diagnostic confirmation if mental-health-related symptoms are identified. Additionally, during the child’s oral health care, professionals should adopt protocols that take into account maternal mental health issues along with each family’s context, making oral health treatment more individualized and effective for children.

## Data Availability

The data used to support the findings of this study can be made available upon request to the corresponding author.
